# Similar decline in mortality rate of older persons with and without type 2 diabetes between 1993 and 2004 the Icelandic population-based Reykjavik and AGES-Reykjavik cohort studies

**DOI:** 10.1186/1471-2458-13-36

**Published:** 2013-01-15

**Authors:** Elin Olafsdottir, Thor Aspelund, Gunnar Sigurdsson, Rafn Benediktsson, Bolli Thorsson, Tamara B Harris, Lenore J Launer, Gudny Eiriksdottir, Vilmundur Gudnason

**Affiliations:** 1 Icelandic Heart Association Research Institute, Kopavogur, Iceland; 2 Public Health Sciences, University of Iceland, Reykjavik, Iceland; 3 Faculty of Medicine, University of Iceland, Reykjavik, Iceland; 4 Landspitali University Hospital, Reykjavik, Iceland; 5 Intramural Research Program, National Institute on Aging, Bethesda, MD, USA

**Keywords:** Cohort study, Type 2 diabetes, Older persons, Cardiovascular disease mortality, Reykjavik study, AGES-Reykjavik

## Abstract

**Background:**

A decline in mortality rates due to cardiovascular diseases and all-cause mortality has led to increased life expectancy in the Western world in recent decades. At the same time, the prevalence of type 2 diabetes, a disease associated with a twofold excess risk of cardiovascular disease and mortality, has been increasing. The objective of this study was to estimate the secular trend of cardiovascular and all-cause mortality rates in two population-based cohorts of older persons, with and without type 2 diabetes, examined 11 years apart.

**Methods:**

1506 participants (42% men) from the population-based Reykjavik Study, examined during 1991–1996 (median 1993), mean age 75.0 years, and 4814 participants (43% men) from the AGES-Reykjavik Study, examined during 2002–2006 (median 2004), mean age 77.2 years, age range in both cohorts 70–87 years. The main outcome measures were age-specific mortality rates due to cardiovascular disease and all causes, over two consecutive 5.7- and 5.3-year follow-up periods.

**Results:**

A 32% decline in cardiovascular mortality rate and a 19% decline in all-cause mortality rate were observed between 1993 and 2004. The decline was greater in those with type 2 diabetes, as illustrated by the decline in the adjusted hazard ratio of cardiovascular mortality in individuals with diabetes compared to those without diabetes, from 1.88 (95% CI 1.24-2.85) in 1993 to 1.46 (95% CI 1.11-1.91) in 2004. We also observed a concurrent decrease in major cardiovascular risk factors in both those with and without diabetes. A higher proportion of persons with diabetes received glucose-lowering, hypertensive and lipid-lowering medication in 2004.

**Conclusions:**

A decline in cardiovascular and all-cause mortality rates was observed in older persons during the period 1993–2004, in both those with and without type 2 diabetes. This decline may be partly explained by improvements in cardiovascular risk factors and medical treatment over the period studied. However, type 2 diabetes still persists as an independent risk factor for cardiovascular mortality.

## Background

Life expectancy has increased in the Western world over recent decades, due in part to a decline in cardiovascular and all-cause mortality rates [1]. However, there has been a concurrent increase in the prevalence of type 2 diabetes [2,3], and the well-established twofold excess risk for coronary heart disease, stroke and mortality associated with diabetes [4] has led to the question of whether those with type 2 diabetes have also benefited from the general decline in mortality risk over recent decades.

Several studies on the secular change in mortality rate in recent decades have shown improved life expectancy in both those with and without type 2 diabetes. In the Hunt Study carried out in Norway a strong general reduction in mortality due to coronary heart disease was observed between 1984 and 1997, but the more than twofold higher mortality rate associated with diabetes persisted over the same period [5]. Similar results were found in the annual Swedish Living Conditions Survey conducted between 1980 and 2004 [6]. Based on the three consecutive NHANES studies between 1971 and 2000, Gregg and co-workers concluded that reduced mortality rates among persons with diabetes had been limited to men [7]. In a population-based study conducted at the Mayo Clinic during 1970–1994, mortality rates for those with and without diabetes declined by 13.8% and 21.4%, respectively, although the mortality burden associated with diabetes increased significantly [8], probably due to an increasing incidence of diabetes. In the most recent study by Gregg and co-workers based on data from the US National Health Interview Survey, death rates in both men and women with diabetes were found to decline substantially between 1997 and 2006, reducing the absolute difference between adults with and without diabetes [9]. Results from the United Kingdom General Practice Research Database likewise showed a decline in relative mortality, that is, the ratio of mortality rates in study and reference populations, for those diagnosed with type 2 diabetes in both men and women in the UK between 1996 and 2006 [10]. The decline in mortality parallels the improvement in levels of cardiovascular risk factors [11], together with improvements in the quality of care and medical treatment for patients with diabetes [12].

In a study on mortality due to coronary heart disease associated with type 2 diabetes in Iceland over the period 1967–1991, the relative risk of death at a mean age of 55 years was found to be 2.0 for men and 2.4 for women, compared to those without diabetes [13]. In the present study, we compared the total and cardiovascular mortality rates in two population-based cohorts of older persons, examined 11 years apart, to determine whether those with type 2 diabetes had fared similarly to those without diabetes. The two cohorts were taken from the population-based Reykjavik Study (RS) and the Age, Gene/Environment Susceptibility-Reykjavik (AGES-Reykjavik) Study, in which changes in cardiovascular risk factors, treatment modalities and mortality rates are monitored.

## Methods

### Study population

In brief, the Reykjavik Study is a population-based cohort study established in 1967 by the Icelandic Heart Association as a long-term prospective cardiovascular survey. Out of a random sample of 30 795 men and women, born from 1907 to 1935 and living in Reykjavik in December 1966, 27 281 were invited to participate, while the remaining 3514 were used as a reference group. A total of 19 381 participated between the years 1967 and 1996, resulting in a 71% recruitment rate. The study sample was divided into six groups and examined in six consecutive stages, as described in detail elsewhere [14,15]. In this study, we report results only from the last stage of the Reykjavik Study (N = 1607), including 1506 older individuals, with an age span of 70–87 years. In the AGES-Reykjavik Study [16], 5764 survivors of the original cohort who had previously participated in the Reykjavik Study were re-examined and 4814 fell within the defined age span. Almost 500 of these (494) had also participated in the last stage of the Reykjavik Study. The first cohort, consisting of 1506 participants (42% men), mean age 75.0 years, was examined between 1991 and 1996 (median 1993), with a median follow-up period of 5.7 years, until mid-2000. The median year of birth was 1919 (inter-quartile range 1917–1921). The second cohort, examined 11 years later, consisting of 4814 participants (43% men), mean age 77.2 years, was examined between 2002 and 2006 (median 2004), with a median follow-up period of 5.3 years, until the end of 2009. The median year of birth was 1927 (inter-quartile range 1923–1931). Informed consent was obtained from all participants.

As part of the baseline examination in both cohorts, a comprehensive questionnaire was administered. The Reykjavik Study participants were asked specific questions about medication, while participants in the AGES-Reykjavik Study were asked to bring all medication and supplements used during the previous 2 weeks to the clinic to be recorded. Participants not completing the questionnaire about diabetes (RS: 2; AGES: 53) and those with incomplete data for other study variables (RS: 97; AGES: 47) were excluded.

The criteria used for the diagnosis of type 2 diabetes in both cohorts were fasting serum glucose of ≥7 mmol/l upon arrival at the clinic, based on the WHO recommendations from 1999 [17], self-reported diabetes in the questionnaire and/or use of diabetes medication. Participants reporting the onset of diabetes before the age of 40 (RS: 2; AGES: 7) were not included to eliminate possible individuals with type 1 diabetes from the study.

Blood samples were drawn after overnight fasting. In the Reykjavik Study, total cholesterol, HDL cholesterol, triglycerides and glucose were analysed on a COBAS Mira instrument. HbA1c was not analysed in the Reykjavik Study. In the AGES-Reykjavik Study, total cholesterol, HDL cholesterol, triglycerides, C-reactive protein (CRP), glucose and HbA1c were analysed on a Hitachi 912 instrument. Both instruments and reagents were from Roche Diagnostics and analyses performed according to the manufacturer’s instructions.

Blood pressure (BP) was measured with a mercury sphygmomanometer with a large cuff, and the mean value of two consecutive blood pressure measurements was used in the analysis. Height and weight were measured and body mass index (BMI) calculated (kg/m^2^).

Information on cause of death was based on data from a complete adjudicated registry of cause of death available from the Icelandic National Roster [18]. Mortality due to cardiovascular disease was defined according to ICD 9–10 (defined as in the SCORE project [19]). In the present study, the duration of an individual’s exposure to risk was calculated from the date of participation in the baseline survey until the date of death from cardiovascular disease or from all causes, or until the end of the follow-up period in each of the two cohorts. Prevalent coronary heart disease was defined as a history of myocardial infarction (ICD 9–10: 410 and I21), percutaneous coronary intervention or coronary artery bypass grafting (coded according to the NOMESCO classification for Surgical Procedures) and was obtained from hospital records, collected from National Health Service Records by the Icelandic Heart Association.

The study was approved by the National Bioethics Committee in Iceland (VSN: 00–063), as well as the Institutional Review Board of the Intramural Research Program of the National Institute on Aging and the Data Protection Authority in Iceland.

### Statistical analyses

The baseline characteristics of the participants from the two cohorts were compared by sex and diabetic status, using either linear or logistic regression with age adjustment. Skewed variables were log-transformed. The Cox proportional hazards regression model was used to estimate hazard ratios and mortality rates using 2-way interaction terms between sex, period, and type 2 diabetes status. Time since entering the study was used as the time scale. In addition to the interaction terms, adjustment was made for age, hypertensive and lipid-lowering medication, prevalent coronary heart disease and for cardiovascular risk factors: total cholesterol, HDL cholesterol, systolic blood pressure, BMI, triglycerides and smoking history. The mortality rate was estimated from the cumulative hazard function after 5 years’ follow-up, and expressed as the rate per 1000 person years. Using interactions allows separate estimates of mortality rates and hazard ratios by sex, diabetes status and study period from the same model and also overall results by weighing together the appropriate contrasts. The proportionality assumption for the hazard ratio associated with type 2 diabetes was inspected graphically and analysed as a time-dependent covariate by testing the significance of the interaction of type 2 diabetes status with the logarithm of the follow-up of time. Significance testing was two-sided and based on a 5% probability level.

When comparing estimates between cohorts, all p-values and confidence intervals were reported from bootstrap estimates based on 200 replications (using independent sampling stratified by cohort) as 494 participants in the Reykjavik Study were alive and participated in the AGES-Reykjavik Study. Naïve estimates of differences between cohorts (treating samples as independent), may then lead to too narrow confidence bands. These estimates are also given for comparison. Sensitivity analysis was also performed by removing overlapping participants (N = 494) from the AGES-Reykjavik cohort. The results were not affected by removing the overlapping participants and are not shown. The change in mortality rate between 1993 and 2004, based on official data from Statistics Iceland, was estimated by applying a Poisson regression model to the age-specific number of deaths for the age range 70 to 87. The model included age, age^2^, sex and a binary indicator for the two periods. The number of alive individuals (mid-year) divided according to age and sex was used as a log offset. The data were analysed using SAS/STAT® software, version 9.2 and Stata version 12.

## Results

### Changes in cardiovascular risk factors and medication between 1993 and 2004

A significant decrease was observed in the levels of the major cardiovascular risk factors (total cholesterol, triglycerides, systolic blood pressure and smoking) between the study periods in both men and women, together with a significant increase in HDL cholesterol, BMI and hypertensive and lipid-lowering medication. The cohorts were then divided into persons with and without diabetes. Individuals with type 2 diabetes had consistently higher baseline levels of triglycerides, BMI and systolic blood pressure at both time points than those without diabetes. Total cholesterol levels, however, were lower in those with type 2 diabetes, significantly so in the AGES cohort, as can be seen in Table 1.

**Table 1:** Baseline characteristics of the older men and women with (T2D Yes) and without type 2 diabetes (T2D No) included in this study

	**Men**				**Women**			
**Variable**	**Reykjavik Study**		**AGES-Reykjavik**		**Reykjavik Study**		**AGES-Reykjavik**	
	**1993**		**2004**		**1993**		**2004**	
**Mean ± SD, IQR or%**	**T2D No**	**T2D Yes**	**T2D No**	**T2D Yes**	**T2D No**	**T2D Yes**	**T2D No**	**T2D Yes**
Number	530	106	1738	330	792	78	2471	275
Age, years	74.7 (±3.5)	74.1 (±3.3) *	77.0 (±4.5)	77.1 (±4.4)	75.3 (±3.6)	75.7 (±4.1)	77.3 (±4.7)	77.9 (±4.5)*
Total cholesterol, mmol/l	6.01 (±1.05)	5.91 (±1.12)	5.23 (±1.07)	4.92 (±1.12) ***	7.03 (±1.25)	6.97 (±1.18)	6.00 (±1.10)	5.53 (±1.13) ***
HDL cholesterol, mmol/l	1.14 (±0.31)	1.05 (± 0.35) *	1.42 (±0.40)	1.25 (±0.32) ***	1.52 (±0.41)	1.34 (±0.47) ***	1.74 (±0.44)	1.52 (±0.43) **
TG mmol/l, median (IQR)	1.07 (±0.61)	1.43 (1.05) ***	0.98 (0.57)	1.30 (0.88) ***	1.21 (0.66)	1.80 (1.30) ***	1.05 (0.64)	1.40 (0.84) ***
CRP mg/l, median (IQR)	-	-	1.80 (2.60)	1.90 (2.70)	-	-	1.90 (2.90)	2.80 (4.60)
BMI, kg/m^2^	25.9 (±3.6)	27.5 (±3.9) ***	26.5 (±3.7)	28.4 (±4.1) ***	26.2 (±4.5)	29.1 (±6.1) ***	26.9 (±4.7)	29.5 (±5.2) ***
Systolic BP, mm Hg	150 (±22)	159 (± 26) **	142 (±20)	145 (±21) *	148 (±21)	152 (±19)	142 (±21)	144 (±21)
Diastolic BP, mm Hg	85 (±10)	88 (± 11) **	76 (±11)	75 (±11) **	80 (±10)	81 (± 11)	72 (± 9)	70 (±10) **
Hypertension (%)^a^	79.2	85.8	77.8	91.1	75.0	85.9	81.3	91.9 ***
Hypertensive medication (%)	26.8	35.8	59.2	79.7***	34.8	48.7*	63.8	83.8 ***
Lipid-lowering medication (%)	1.9	2.8	27.3	37.7	2.0	0	17.7	37.0***
Prevalence of CHD (%)^b^	17.4	17.0	24.8	31.8**	4.6	5.1	8.3	14.0*
Prevalence of MI (%)^c^	13.4	13.2	12.4	14.6	3.9	3.9	4.6	7.6
Family history of MI (%)	26.0	21.7	34.1	37.7**	29.3	32.1	42.6	49.8 **
Current smoker (%)	17.6	20.8	11.0	9.2	17.6	11.5	12.5	9.8
Haemoglobin A1c (%)	-	-	5.55 (±0.31)	6.44 (±0.83) ***	-	-	5.61 (±0.32)	6.42 (±0.88) ***
Glucose, mmol/l	5.77 (±0.48)	8.16 (±2.01) ***	5.58 (±0.51)	7.91 (±2.16) ***	5.51 (±0.56)	7.79 (±2.24) ***	5.43 (±0.51)	7.73 (±2.09) ***

Significance estimates: *p < .05; **p < .01; *** p < .001 for age-adjusted comparison between the Reykjavik Study and the AGES- Reykjavik Study.

^a^Hypertension, those with systolic BP > 140 mmHg, diastolic BP > 90 mm Hg or on hypertensive medication.

^b^Prevalence of CHD, from hospital records, of those with history of myocardial infarction (MI), percutaneous coronary intervention, and coronary-artery bypass grafting.

^c^Prevalence of MI, from hospital records, of those with history of MI.

BMI: body mass index; BP: blood pressure; CHD: coronary heart disease; CRP: C-reactive protein; TG: triglyceride.

In 1993 the prevalence of coronary heart disease, obtained from hospital records, was similar in those with and without type 2 diabetes for both sexes, i.e., about 17% in men and 5% in women. In 2004, however, the prevalence of coronary heart disease in those with diabetes had risen to 32% in men and 14% in women, compared to 25% in men and 8% and women without diabetes. During this period, little change was seen in the prevalence of myocardial infarction, except in women with diabetes, where almost twice as many had experienced a myocardial infarction in 2004 than in 1993 (Table 1).

An increase in hypertensive medication observed between 1993 and 2004 was more marked in individuals with diabetes than in those without, although increases were seen in all groups (Table 1). Over the same period, the observed decrease in mean values of systolic blood pressure was greater in men and women with diabetes than in those without (Table 1).

Use of statins was not reported in the 1993 study, while other lipid-lowering medication was used by 3% of individuals with known diabetes and by under 2% of all participants. In 2004 about 44% of individuals with known diabetes were taking statins, but only 27% of the men and 18% of the women without diabetes were statin users. Individuals using statins had 1.26 (95% CI 1.19-1.32) mmol/l lower mean total cholesterol levels, irrespective of their diabetic status.

The prevalence of type 2 diabetes was about the same in both cohorts, as can be seen in Table 2: 16.7% and 16.0% in men and 9.0% and 10.0% in women, in 1993 and 2004, respectively. The proportion of individuals already diagnosed as having type 2 diabetes at baseline increased from 41% in 1993 to 69% in 2004, as did the proportion of individuals already diagnosed and receiving glucose-lowering medication. In 1993 only 59% of men with prior diagnosis of type 2 diabetes were on glucose-lowering medication, compared to 76% in 2004. Similarly, 31% of women diagnosed with type 2 diabetes in 1993 were on glucose-lowering medication compared to 70% in 2004 (Table 2).

**Table 2:** Prevalence of type 2 diabetes (T2D) and glucose-lowering treatment in the two study cohorts

**Reykjavik Study 1991–1996 (median 1993)**	**Men**	**Women**	**Men and women**	**% of total T2D**
	**N = 636**	**N = 870**	**N = 1506**	
Total T2D, prevalence % (n)	16.7 (106)	9.0 (78)	12.2 (184)	100
Diagnosed at baseline % (n)	10.5 (67)	4.8 (42)	7.2 (109)	59
With prior T2D diagnosis % (n)	6.1 (39)	4.1 (36)	5.0 (75)	41
Mean T2D duration in years (±SD)			10.1 (±9.4)	
Glucose-lowering treatment in patients with prior T2D diagnosis				
% on glucose-lowering medication (n)	59 (23)	31 (11)		
% on special diet only (n)	41 (16)	69 (25)		
**AGES-Reykjavik 2002–2006 (median 2004)**	**Men**	**Women**	**Men and women**	**% of total T2D**
	**N = 2068**	**N = 2746**	**N = 4814**	
Total T2D, prevalence % (n)	16.0 (330)	10.0 (275)	12.6 (605)	100
Diagnosed at baseline % (n)	4.7 (97)	3.0 (83)	3.7 (180)	31
With prior T2D diagnosis % (n)	11.3 (233)	7.0 (192)	8.8 (425)	69
Mean T2D duration in years (±SD)			10.7 (±10.0)	
Glucose-lowering treatment in patients with prior T2D diagnosis				
% on glucose-lowering medication (n)	76 (161)	70 (112)		
% on special diet only (n)	24 (52)	30 (48)		

### Changes in cardiovascular and all-cause mortality rates between 1993 and 2004

**Figure 1:**
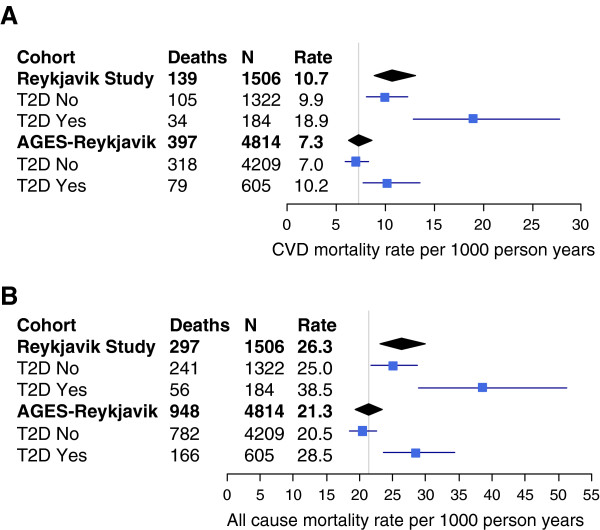
**A) Cardiovascular disease (CVD) mortality rate and B) all-cause mortality rate per 1000 person years for older individuals with (T2D Yes) and without type 2 diabetes (T2D No).** Rates were adjusted to age 75, sex and the mean levels of cardiovascular risk factors (total cholesterol, HDL cholesterol, systolic blood pressure, BMI, triglycerides and smoking history), and hypertensive and statin medication within each cohort. In the Reykjavik Study (1993) the median follow-up period was 5.7 years and in the AGES-Reykjavik Study (2004) 5.3 years. The vertical lines represent the mortality rate for the AGES-Reykjavik cohort (N = 4814). The horizontal lines show 95% confidence intervals.

Between the last stage of the Reykjavik Study (1993) and the AGES-Reykjavik Study (2004), the secular decline in cardiovascular mortality rates observed in older individuals (aged 70–87 years) was 32% (95% CI 14%-45%) and in all-cause mortality rates 19% (95% CI 6%-30%), as illustrated in Figure 1. Rates were adjusted to age 75, sex (male 43%), the mean levels of cardiovascular risk factors (total cholesterol, HDL cholesterol, systolic blood pressure, BMI, triglycerides and smoking history), and hypertensive and statin medication within each cohort.

To evaluate the contribution of changes in the level of risk factors and medication between study periods, the mortality rates were compared after adjusting for the common mean values of the these factors over cohorts. Moderate attenuation was seen and the decline estimated to be 25% (95% CI 3%-42%, bootstrap 2%-43%) for cardiovascular mortality and 16% (95% CI 1%-29%, bootstrap 2%-28%) for all-cause mortality.

When the decline in mortality was estimated for those with and without type 2 diabetes, both cardiovascular (Figure 1A) and all-cause mortality rates (Figure 1B) declined similarly over the approximately 11 years between the two studies. The observed decline was greater in individuals with type 2 diabetes than in those without, albeit not statistically significant. From 1993 to 2004, a decline of 46% (95% CI 17% -65%) in cardiovascular mortality rate was seen in those with diabetes; the rate falling from 18.9 to 10.2 per 1000 person years. For individuals without diabetes the decrease in cardiovascular mortality rate was 29% (95% CI 10%-45%); the rate decreasing from 9.9 to 7.0 per 1000 person years over the same period. The reduction observed in all-cause mortality rate in those with type 2 diabetes was 27% (95% CI −10%-46%) and in those without type 2 diabetes 18% (95% CI 4%-30%). Interactions between sex and the effect of diabetes status were neither significant for cardiovascular mortality (p = 0.41) nor total mortality (p = 0.51).

The hazard ratios for the relative risk of death from cardiovascular disease with respect to diabetes were estimated (Table 3). The hazard ratios adjusted for age and sex, fell from 2.21 (95% CI 1.46-3.34) in the Reykjavik Study (1993) to 1.69 (95% CI 1.30-2.20) in the AGES-Reykjavik Study (2004). When additionally adjusted for cardiovascular risk factors, they fell from 1.88 (95% CI 1.24-2.85) in 1993 to 1.46 (95% CI 1.11-1.91) in 2004. No further attenuation was seen after additional adjustment for surgical treatment (percutaneous coronary intervention and coronary-artery bypass grafting), or hypertensive and statin medication.

**Table 3:** Hazard ratios (HR) with 95% confidence intervals (CI) for the relative risk of death. Cardiovascular disease (CVD) mortality and all-cause mortality in individuals with type 2 diabetes (T2D) compared to those without in each cohort

	**Adjusted for age and sex**		**Adjusted for age, sex, and CVD risk factors**^**a**^		**Adjusted for age, sex, CVD risk factors**^**a**^**, surgical intervention**^**b**^**, HTNmed.**^**c**^		**Adjusted for age, sex, CVD risk factors **^**a**^**, surgical intervention**^**b**^**, HTNmed.**^**c**^**and statin use**	
	**HR**	**95% CI**	**HR**	**95% CI**	**HR**	**95% CI**	**HR**	**95% CI**
**Death from CVD**								
Reykjavik Study: with T2D vs. without T2D	**2.21**	1.46-3.34	**1.88**	1.24-2.85	**1.89**	1.25-2.87	**1.89**	1.24-2.86
AGES-Reykjavik: with T2D vs. without T2D	**1.69**	1.30-2.20	**1.46**	1.11-1.91	**1.46**	1.12-1.92	**1.47**	1.12-1.93
**Death from all causes**								
Reykjavik Study: with T2D vs. without T2D	**1.66**	1.23-2.25	**1.55**	1.14-2.11	**1.56**	1.15-2.11	**1.54**	1.13-2.09
AGES-Reykjavik: with T2D vs. without T2D	**1.47**	1.23-1.75	**1.38**	1.15-1.65	**1.38**	1.15-1.65	**1.39**	1.16-1.67

^a^CVD risk factors: total cholesterol, HDL cholesterol, systolic blood pressure, BMI, triglycerides and smoking history.

^b^Surgical intervention: percutaneous coronary intervention and coronary-artery bypass grafting.

^c^HTNmed: hypertensive medication.

The hazard ratios for relative risk of death from all causes declined from 1.66 (95% CI 1.23-2.25) in 1993 to 1.47 (95% CI 1.23-1.75) in 2004 when adjusted for age and sex. After additional adjustment for cardiovascular risk factors, the hazard ratio decreased from 1.55 (95% CI 1.14-2.11) to 1.38 (95% CI 1.15-1.65). This 11% (bootstrap 95% CI −26%-37%, naïve 95% CI −24%-37%) decline in hazard ratios did not reach statistical significance.

The hazard ratios were also estimated after omitting data from participants who attended during both study periods. The hazard ratios for the relative risk of death from cardiovascular disease, adjusted for age and sex, fell from 2.15 (95% CI 1.41 - 3.27) in the Reykjavik Study (1993) to 1.58 (95% CI 1.17 - 2.13) in the AGES-Reykjavik Study (2004). When additionally adjusted for cardiovascular risk factors, the hazard ratios fell from 1.82 (95% CI 1.19 - 2.78) in the Reykjavik Study to 1.34 (95% CI 0.98 - 1.83) in the AGES-Reykjavik Study.

The hazard ratios for relative risk of death from all causes declined from 1.63 (95% CI 1.20 - 2.21) in 1993 to 1.41 (95% CI 1.15 - 1.72) in 2004 when adjusted for age and sex. After additional adjustment for cardiovascular risk factors, the hazard ratio decreased from 1.51 (95% CI 1.10 - 2.06) to 1.30 (95% CI 1.06 - 1.60). This 14% (bootstrap 95% CI −26% - 40%, naïve 95% CI −22% - 39%) decline in mortality ratio did not reach statistical significance.

## Discussion

In this population-based cohort study we observed a decline in mortality rates of older individuals between the two study periods, together with favourable changes in risk factors and an increase in medical treatment. The observed decline in the cardiovascular and all-cause mortality rates in our study are comparable, according to Statistics Iceland [18], to the decline seen between 1993 and 2004 for the same age group in the Icelandic population at large, of 36% (95% CI 25%-45%) in cardiovascular, and 21% (95% CI 14%-28%) in all-cause mortality rates. As the decline in coronary heart disease mortality over recent decades has been attributed to a reduction in cardiovascular risk factors [20] we adjusted for mean values of risk factors and medical treatment over the two cohorts and found that the estimated cardiovascular mortality rate fell from 32% to 25%, indicating that the decline in mortality rate is only partly due to a reduction in cardiovascular risk factors and increase in medical treatment, and therefore remains to be fully explained.

The decline in both cardiovascular and all-cause mortality rates observed was somewhat greater in individuals with diabetes than in those without, although the difference did not reach statistical significance. The age- and sex-adjusted hazard ratio for cardiovascular mortality declined from 2.21 in 1993 to 1.69 in 2004, and for all-cause mortality, from 1.66 to 1.47. Compared to the estimated relative risk of death from all causes, between 1967 and 1991, of 1.9 for men and 1.7 for women with type 2 diabetes, in a middle-aged Icelandic cohort [13], the hazard ratio for death from all causes in the cohort of older individuals from 1993 is only slightly lower. The low number of participants in the 1993 cohort, however, does not allow a detailed study on the effect of either disease duration or the difference between men and women, so the comparison must be made with caution.

The observed attenuation in hazard ratio after adjusting for cardiovascular risk factors did not alter the trend in hazard ratio reduction between the two time points, emphasizing that the increased mortality rate of those with type 2 diabetes persisted over time, and was independent of cardiovascular risk factors. Our results are thus in agreement with those from a collaborative meta-analysis of 102 prospective studies, that conventional cardiovascular risk factors do not explain why twice the incidence of cardiovascular diseases is seen in those with type 2 diabetes than in those without diabetes [4].

It has been shown that the increased mortality risk for persons with type 2 diabetes is related to both age at onset and duration of disease. In an observational study from Tayside, Scotland, Barnett and co-workers showed that hazard ratios for mortality decreased with increasing age at diagnosis, and increased mortality risk was not evident until 2 years after diagnosis [21]. There was a trend towards increasing risk with increasing duration of disease, which started to decline 8 years after diagnosis. In our study the mean duration of disease was just over 10 years in both cohorts, but the percentage of individuals diagnosed at baseline was much higher in the cohort from 1993, i.e. 59% compared to 41% in 2004, so the mean duration of disease until the end of follow-up was lower in 1993 than in 2004. Increased awareness of the risk of diabetes in old age, both among professionals and the general public, may be the reason for the increase in diagnosed diabetes at entry in the AGES-Reykjavik cohort, and those in the Reykjavik Study cohort may, therefore, have had untreated disease for some time. The possible over diagnosis using only one glucose measurement [22] for newly diagnosed diabetes, may have led to both a greater dilution bias and a shorter mean duration of disease in the Reykjavik Study cohort, and thus underestimation of the mortality rate of diabetic persons in that cohort.

In the Northern Sweden Monica Study [23] it was observed that long-term survival after a first myocardial infarction in middle age was markedly lower in diabetic patients than in those without diabetes. Similarly, the long-term survival after the first stroke was much lower in diabetic patients than in those without diabetes [24], although survival did improve throughout the period from 1985 to 2005. The decline in relative mortality due to diabetes seen between 1996 and 2006 in the UK [10] possibly resulted from both improved trends in the incidence of and mortality from cardiovascular disease and improved medical attention. In Denmark reduced rates of death and cardiovascular disorders were observed in patients with type 2 diabetes in the Steno-2 Study using intensive intervention with multiple drug combinations [25]. Although a general trend towards improved survival of patients with diabetes compared with those without diabetes is illustrated in these studies the survival gap still persists.

Another finding of our study was the increase in recorded history of acute cases of coronary heart disease seen between the two periods, in both those with and without diabetes. This is probably due to the increased survival rate of coronary heart disease patients, following the favourable changes in cardiovascular risk factor levels in all age groups, possibly resulting in milder disease, but an increase in hospital referral between study periods cannot be excluded. More intensive medical intervention has also tended to reduce premature deaths from coronary heart disease, especially from myocardial infarction, as was observed in Iceland during the period 1981 to 2006 in the age group of 25–74 years [20].

Regarding the changes in medication over the study period, we have shown in a previous study that statin use, irrespective of glucose-lowering or antihypertensive medication, is associated with lower mortality rate of older individuals with type 2 diabetes compared to those without diabetes [26]. In 1993 statin therapy had not been introduced and their general use in 2004 could explain some of the drop in mortality rate observed between the study periods.

The Icelandic population is comparable to other Western populations with respect to cardiovascular morbidity and mortality [27], and the low prevalence of diabetes in Iceland has been changing similarly to trends in other Western societies [28]. Our data show that the life expectancy of older individuals diagnosed with type 2 diabetes has increased concomitantly with that of the population in general, and that reductions in cardiovascular risk factors and improved treatment modalities may have benefited them at least as much as those without diabetes.

The strengths of the present study are the proportionally large national representation of older individuals in this population-based study, the high participation rate and the comprehensive information available on morbidity and mortality. It was observed that frailer individuals participated to a lesser extent, causing a possible bias, but non-attendees in this study have been shown to have comparable levels of conventional cardiovascular risk factors [16] during earlier visits. A weakness of this study is that the diagnosis of diabetes at baseline was based on a single measurement of fasting glucose (≥ 7 mmol/l), thereby possibly causing a positive bias in the number of persons diagnosed at entry [22]; this may also have caused a dilution bias in the estimates of mortality rates. Those developing diabetes after having answered the questionnaire will have been missed, which may have caused a slight bias in the mortality estimates. The power of the data was not enough to make stratifying for sex statistically significant and may be listed as a weakness. Prevalent coronary heart disease only included acute myocardial infarction cases and coronary procedures. Accordingly, subjects with angina were not included, causing a possible classification bias.

## Conclusions

We observed a decline in cardiovascular and all-cause mortality rates in two population-based cohorts of older individuals, with and without type 2 diabetes during the period 1993 to 2004. A decrease in the levels of major cardiovascular risk factors, together with improved diagnosis of diabetes and better medical treatment, is usually the explanation given for the decline in mortality rate seen in study populations, and hence the general population. The present study, however, shows that type 2 diabetes still persists as an independent risk factor for cardiovascular and all-cause mortality, also in old age.

## Abbreviations

AGES or AGES-Reykjavik: Age Gene/Environment Susceptibility - Reykjavik Study; BMI: Body mass index; BP: Blood pressure; CHD: Coronary heart disease; CRP: C-reactive protein; CVD: Cardiovascular disease; HbA1c: Haemoglobin A1c; ICD-9 and ICD-10: International Statistical Classification of Diseases and Related Health Problems 9^th^ and 10^th^ Revision; IQR: Inter-quartile range; MI: Myocardial infarction; RS: Reykjavik Study; SD: S deviation; TG: Triglycerides; WHO: World Health Organization.

## Competing interests

The authors declare that they have no competing interests.

## Authors’ contributions

EO, TA and VG drafted the manuscript. EO and TA performed the statistical analysis. Data collection and preparation were performed by EO, TA, VG, BT, GE, GS, LJL and TBH. All authors contributed to the interpretation of the findings, and read and approved the final manuscript.

## Pre-publication history

The pre-publication history for this paper can be accessed here:

http://www.biomedcentral.com/1471-2458/13/36/prepub

## References

[B1] Kesteloot H Sans S Kromhout D Dynamics of cardiovascular and all-cause mortality in Western and Eastern Europe between 1970 and 2000 Eur Heart J 2006 27 1 107 13 16204263 10.1093/eurheartj/ehi511

[B2] Zimmet P Alberti KG Shaw J Global and societal implications of the diabetes epidemic Nature 2001 414 6865 782 7 10.1038/414782a 11742409

[B3] Lam DW Leroith D The worldwide diabetes epidemic Curr Opin Endocrinol Diabetes Obes 2012 19 2 93 6 22262000 10.1097/MED.0b013e328350583a

[B4] Sarwar N Gao P Seshasai SR Gobin R Kaptoge S Di Angelantonio E Diabetes mellitus, fasting blood glucose concentration, and risk of vascular disease: a collaborative meta-analysis of 102 prospective studies Lancet 2010 375 9733 2215 22 10.1016/S0140-6736(10)60484-9 20609967 PMC2904878

[B5] Dale AC Vatten LJ Nilsen TI Midthjell K Wiseth R Secular decline in mortality from coronary heart disease in adults with diabetes mellitus: cohort study BMJ 2008 337 a236 10.1136/bmj.39582.447998.BE 18595902 PMC2453302

[B6] Eliasson M Talback M Rosen M Improved survival in both men and women with diabetes between 1980 and 2004–a cohort study in Sweden Cardiovasc Diabetol 2008 7 32 10.1186/1475-2840-7-32 18937871 PMC2586621

[B7] Gregg EW Gu Q Cheng YJ Narayan KM Cowie CC Mortality trends in men and women with diabetes, 1971 to 2000 Ann Intern Med 2007 147 3 149 55 10.7326/0003-4819-147-3-200708070-00167 17576993

[B8] Thomas RJ Palumbo PJ Melton LJ 3rd Roger VL Ransom J O’Brien PC Leibson CL Trends in the mortality burden associated with diabetes mellitus: a population-based study in Rochester, Minn, 1970–1994 Arch Intern Med 2003 163 4 445 51 10.1001/archinte.163.4.445 12588203

[B9] Gregg EW Cheng YJ Saydah S Cowie C Garfield S Geiss L Barker L Trends in death rates among U.S. Adults with and without diabetes between 1997 and 2006: findings from the national health interview survey Diabetes Care 2012 35 6 1252 7 10.2337/dc11-1162 22619288 PMC3357247

[B10] Gulliford MC Charlton J Is relative mortality of type 2 diabetes mellitus decreasing? Am J Epidemiol 2009 169 4 455 61 19037005 10.1093/aje/kwn342

[B11] Ford ES Trends in the risk for coronary heart disease among adults with diagnosed diabetes in the U.S.: findings from the National Health and Nutrition Examination Survey, 1999–2008 Diabetes Care 2011 34 6 1337 43 10.2337/dc10-2251 21505207 PMC3114334

[B12] Saaddine JB Cadwell B Gregg EW Engelgau MM Vinicor F Imperatore G Narayan KM Improvements in diabetes processes of care and intermediate outcomes: United States, 1988–2002 Ann Intern Med 2006 144 7 465 74 10.7326/0003-4819-144-7-200604040-00005 16585660

[B13] Vilbergsson S Sigurdsson G Sigvaldason H Sigfusson N Coronary heart disease mortality amongst non-insulin-dependent diabetic subjects in Iceland: the independent effect of diabetes. The Reykjavik Study 17-year follow up J Intern Med 1998 244 4 309 16 10.1046/j.1365-2796.1998.00368.x 9797494

[B14] Jonsdottir LS Sigfusson N Sigvaldason H Thorgeirsson G Incidence and prevalence of recognised and unrecognised myocardial infarction in women. The Reykjavik Study Eur Heart J 1998 19 7 1011 8 10.1053/euhj.1998.0980 9717035

[B15] Sigurdsson E Thorgeirsson G Sigvaldason H Sigfusson N Prevalence of coronary heart disease in Icelandic men 1968–1986. The Reykjavik Study Eur Heart J 1993 14 5 584 91 10.1093/eurheartj/14.5.584 8508850

[B16] Harris TB Launer LJ Eiriksdottir G Kjartansson O Jonsson PV Sigurdsson G Age, Gene/Environment Susceptibility-Reykjavik Study: multidisciplinary applied phenomics Am J Epidemiol 2007 165 9 1076 87 10.1093/aje/kwk115 17351290 PMC2723948

[B17] World Health Organization (WHO) Definition, diagnosis and classification of diabetes mellitus and its complications: report of a WHO consultation. Part 1, Diagnosis and classification of diabetes mellitus 1999 WHO, Geneva

[B18] Statistics Iceland 2010 http://www.statice.is/Statistics/Population/Births-and-deaths

[B19] Conroy RM Pyorala K Fitzgerald AP Sans S Menotti A De Backer G Estimation of ten-year risk of fatal cardiovascular disease in Europe: the SCORE project Eur Heart J 2003 24 11 987 1003 10.1016/S0195-668X(03)00114-3 12788299

[B20] Aspelund T Gudnason V Magnusdottir BT Andersen K Sigurdsson G Thorsson B Analysing the large decline in coronary heart disease mortality in the Icelandic population aged 25–74 between the years 1981 and 2006 PLoS One 2010 5 11 e13957 10.1371/journal.pone.0013957 21103050 PMC2980472

[B21] Barnett KN Ogston SA McMurdo ME Morris AD Evans JM A 12-year follow-up study of all-cause and cardiovascular mortality among 10,532 people newly diagnosed with Type 2 diabetes in Tayside, Scotland Diabet Med 2010 27 10 1124 9 10.1111/j.1464-5491.2010.03075.x 20854379

[B22] Selvin E Crainiceanu CM Brancati FL Coresh J Short-term variability in measures of glycemia and implications for the classification of diabetes Arch Intern Med 2007 167 14 1545 51 10.1001/archinte.167.14.1545 17646610

[B23] Eliasson M Jansson JH Lundblad D Naslund U The disparity between long-term survival in patients with and without diabetes following a first myocardial infarction did not change between 1989 and 2006: an analysis of 6,776 patients in the Northern Sweden MONICA Study Diabetologia 2011 54 10 2538 43 10.1007/s00125-011-2247-9 21779872

[B24] Eriksson M Carlberg B Eliasson M The disparity in long-term survival after a first stroke in patients with and without diabetes persists: the Northern Sweden MONICA study Cerebrovasc Dis 2012 34 2 153 60 10.1159/000339763 22907276

[B25] Gaede P Lund-Andersen H Parving HH Pedersen O Effect of a multifactorial intervention on mortality in type 2 diabetes N Engl J Med 2008 358 6 580 91 10.1056/NEJMoa0706245 18256393

[B26] Olafsdottir E Aspelund T Sigurdsson G Thorsson B Eiriksdottir G Harris TB Effects of statin medication on mortality risk associated with type 2 diabetes in older persons: the population-based AGES-Reykjavik Study BMJ Open 2011 1 1 e000132 10.1136/bmjopen-2011-000132 22021772 PMC3191423

[B27] Aspelund T Thorgeirsson G Sigurdsson G Gudnason V Estimation of 10-year risk of fatal cardiovascular disease and coronary heart disease in Iceland with results comparable with those of the Systematic Coronary Risk Evaluation project Eur J Cardiovasc Prev Rehabil 2007 14 6 761 8 10.1097/HJR.0b013e32825fea6d 18043296

[B28] Bergsveinsson J Aspelund T Gudnason V Benediktsson R Prevalence of type 2 diabetes mellitus in Iceland 1967 to 2002 Laeknabladid 2007 93 5 397 402 17502682

